# On the Importance of the Teeth, in Reference to the Health

**Published:** 1880-06

**Authors:** M. Bissel

**Affiliations:** Camden, S. C.


### THE DENTAL REGISTER.

VOL. XXXIV.] JUNE, 1880. [No. 6.

### ON THE IMPORTANCE OF THE TEETH, IN REFERENCE TO THE HEALTH.

BY M. BISSEL, CAMDEN, S. C.

When the importance of a serviceable Dental organization is duly considered, it appears to be an unfortunate fatality on the part of a majority of individuals, that the subject should be so indifferently regarded. The *Teeth* are essential to the healthy action of the important functions of the economy; for with them commences the first operation of the process of digestion. The old maxim, that "food well masticated, is half digested," is a trueism; and it will be found entirely useless to expect that "good digestion will wait on appetite, and health on both:" so long as these all important adjuncts are in a diseased condition, the gums unhealthy as a consequence, both imparting their vitiated secretions to the stomach, thereby destroying the normal action of the digestive organs, causing derangement in the system, giving rise to many diseases of a nervous type, to dyspepsia and many other ills, which are formed from this Pandora's box; and it is not improbable that the highly vitiated condition of the mouth may be a factor in causing a *diseased* condition of the *lungs*.

There may be exhibited some degree of temerity, if not of imprudence, on the part of the writer, (who has no claim to any *affix* to his name, either M. D. or D. D. S.) to state such a proposition; yet there may appear to be reasonable grounds for the assertion, when the fact is considered, that we breathe twenty thousand times in twenty-four hours, and take into the lungs two thousand gallons every day, and that the air passing over the accumulation of filth, the mouth contains, carries with it particles of putrid matter to the lungs, and that this continued impact of foreign and diseased matter upon the delicate tissues continues for months and years, and will eventually cause disease in them. That such particles of putrid matter are carried *to* the *lungs*, must be admitted, when every exhalation is charged with it; as is evidenced to the olfactories of all who come in contact with the breath from a mouth so loaded with offensiveness. When a person breathes an atmosphere loaded with the fumes of spirits, or other volatile substances, a portion is taken up by the absorbents of the lungs and carried into the system, and the lungs thus become a ready inlet to contagien, and other deleterious influences diffused through the air which we breathe.

It is held that dyspeptic symptoms are often the first indications of consumption, and that the lungs suffer only after the digestive organs have been for a time diseased. When from defective food or impaired digestion, the blood is impoverished in quality, the lungs speedily suffer: such is found to be the case among the poorly fed; among the higher classes the blood may be impoverished and the lungs injured, for the want of power to perfectly digest the food, and the lungs may be injured from imperfect digestion.

Digestion is a complicated process, not confined alone to the stomach; it begins in the *mouth*, where the food in the process of mastication is subjected to the action of the *saliva*, and this mastication of the food is the only part of digestion which is left to an individual's discretion, and it is the one which is indifferently performed in a majority of cases, and causes many ills. When the food is bolted down, instead of being well masticated, it enters the stomach in lumps of different sizes, not reduced to a soft pulpy condition, on which the gastric juice can promptly act; as a consequence the soft coats and yielding tissues of the stomach are made to do the grinding of the lumps, and the time for digesting the food is lengthened, sowing the seeds of disease.

If the imperfect comminution of the food impairs the perfect action of the digestive organs, may not the abnormal state be charged to the bad condition of the teeth in many instances? A few cases of imperfect mastication and consequent imperfect assimilation of the food, might not have a marked injurious effect; but the continuance of the depraved state of the masticating organs is often for months and years, and must tell unfavorably on organs so sensitive as the lungs.

Dr. McCarrick, a writer on disease of the lungs, maintains that consumption or tubercular disease may be caused from breathing air which has been breathed by man or animals. That the hourly elimination of carbonic acid by the lungs will amount to ten or twelve cubic inches, and if the air is already contaminated with that gas, and other organic effete matter, the power to remove these from the body is severely injured, and the detritus of degeneration being retained becomes tuberculous. By a parity of reasoning may not the breathing continuously of air highly impregnated with particles of diseased matter, be quite as much a cause of disease in the lungs ? In the first case the air breathed is deprived of the constituents necessary to the healthy condition of the lungs ; in the other, the air is impregnated with a poison highly detrimental to the sound and healthy condition of these delicate organs. There must be very fine spun objections offered to condemn the proposition. It is no doubt true that diseased dentine is often caused from constitutional derangements; the effect, and not the cause of failure in the general health, but both conditions are intensified by the depraved condition present in either.

In further support of the proposition, that diseased lungs may be caused from a vitiated condition of the oral cavity, the following may be offered : The *Tartar* and *fluids* in the mouth contain myriads of formations, both animal and vegetable ; a fact, the microscope has abundantly developed, and the use of the microscope has developed many of nature's secrets. In studying the laws of life, physic and surgery has advanced from its aid. Its use has developed the fact, that the pestilences which carry death into our homes are frequently caused by minute animal and vegetable organizations, which enter the system from apparently pure air. May not these minute parasites be also a source of disease to the lungs ? Is it improbable, that these minute animalcules may be carried to the lungs in every breath, and being out of their element, become effete matter in the lung cells ? Their location in the lungs would be abnormal, the mouth being their natural *habitat*, there they increase; when carried to the lungs, they do not prosper, their career, when in their natural element brief at best, ends when out of it, and they become highly injurious accumulations. May not myriads be taken into the stomach with the food, and enter into the circulation and pass with the blood into the lungs, during the airification of that fluid?

The *Trichinia* has recently been found to be a new cause of disease. These minute organisms are supposed to be carried to the stomach with the food, and entering into the circulation cause serious derangements in the economy. This fact may offer reasonable grounds for the assertion that the formations in the mouth can become a source of disease to the lungs. In the hypothesis stated, there are two sources of evil: from direct contact with the lungs, being carried there by the breath, and their presence in the lung cells from passing with the food into the stomach and into the circulation.

One hypothesis in regard to marsh malaria, refers its origin to certain microscopic growths which disseminate and effect a lodgement in the human system, and there set in motion *organic changes*, producing various phenomena of disease. Through what channel do these growths find entrance into the system, if not through the lungs? And the presence of these growths must set in motion "*organic changes*" in the lungs, as well as in other portions of the economy. Is the assertion less tenable in regard to the parasites found in the mouth, and their effect when carried to, and lodged in the lungs?

One theory in regard to the origin of yellow fever, it is held to be caused by a species of fungoid plant, containing myriads of minute cells, which when falling into a suitable fluid, as the blood, have the property of multiplying indefinitely, assuming by a species of evolution, the form of minute crystaline atoms. These cells it is held enter the system through the lungs, and also through the stomach with the food and into the circulation. They multiply in the blood and cause chemical changes therein as ordinary ferments act upon syrups in changing them into carbonic acid and alcohol. But the blood may not always be in a condition to take on this state from the presence of these organisms, and the blood may possess the power of casting them off through the excretions; and it is only when these atoms are in excess, that the fever is fully developed. One atom or a thousand may not excite the disease when the blood is indisposed to the change, or when the system has the power to rapidly excrete the poison.

Is there not a marked correspondence between the organisms in the mouth and their effect on the lungs and the effect of the organisms originating yellow fever upon the system through the lungs? It is not supposed that in every instance of diseased dentine the lungs will necessarily be affected, for the power of resistance in one case may be in advance of another, and failure may arise when one or more of the conditions essential to the propagation of the germs is absent, or when the vitality of the parasites has been in a measure, if not entirely destroyed by disinfection.

Again, take *contagion* as a factor in consumption. Physicians are much less inclined than formerly to underrate contagion as one of the factors causing diseased lungs. Dr. Welch, who has written on the subject, had considered contagion anything but a power, in 1860, but says in 1878: "My belief in the reality of such transmutability has become strengthened; I have met with so many examples of the kind that coincidence becomes itself an explanation difficult of acceptance. It is no proof that consumption is not contagious, because husbands and wives, who have been in close attendance on their deceased consorts, have not always been affected, for the tuberculization process fails occassionally when experiments have been made on animals, and the indirect transmission of *infecting particles* from the lungs of one individual to another, is certainly much more liable to failure."

What are those *infecting particles* referred to as factors in producing consumption, if they are not microscopic organisms thrown off from diseased lungs? They are not particles of normal tissue, but tissue that has become individualized into the organisms referred to, for no sooner does highly organized tissue wane in its vitality, than its constituent particles individualizing themselves give rise to lower forms of life. Admitting the doctors argument in regard to contagion from diseased lungs, is not the ground equally reasonable in regard to the parasites found in the mouth. Both find their objective point in the lungs, and the presence of these growths set in motion organic changes in these delicate tissues.

While many of the medical profession admit the fact that a carious condition of the teeth is a source to which some of "the ills that flesh is heir to" can be traced, yet they are indisposed to the doctrine that this scourge of the human family, consumption, can be originated from diseased teeth, and the vitiated state of the mouth, and, as a consequence, of the digestive organs. The foregoing on the point (and their number might be increased) would appear to offer reasonable grounds for sustaining the proposition that has been advanced. And if there is a modicum of truth in the statements given in regard to the importance of a healthy condition of the teeth and their surroundings, the statements should be enough to place all on their guard in relation to the care and preservation of their dental organs.

As an evidence of the deleterious effect of diseased teeth on the general health, the following case of a lady patient of Dr. Dwinnelle, of New York, may be stated. It is one out of hundreds that could be offered: The case appears to have baffled several physicians The patient had twice visited Europe on account of her health : bronchitis, dyspepsia, with other diseases, and, finally, consumption, were assigned as the cause of her condition. Dr. D., in reporting the case, says: "I saw her after her last return from Europe; found her much emaciated, nervous, with a rapid pulse, no appetite; an exceedingly excoriating and offensive secretion accumulating in her throat. The left eye protruded from its socket; had lost the sense of smell and taste for two years. The nasal passage on the left side was completely closed. I diagnosed disease of the antrum. There were but four teeth in the upper jaw; the second molar was diseased and dead. I passed a probe up beside one of its roots into the antrum, and for a distance of nearly four inches. The molar tooth and all the others were extracted, and the after treatment resulted in the lady's restoration to health."

Other recorded cases of dental diseases that have baffled the practice of some physicians, have been successfully treated where they have been in charge of professional men, whose views were not circumscribed by the dictum of men who have held the profession of the dentist, and dental diseases, as entirely beneath their consideration.

While it is deemed essential to the practice of the successful dentist that he should possess a knowlege of medicine and surgery, it might with some propriety be impressed upon the minds of a few at least of the medical profession, the advantage in their general practice to possess some acquaintance with dental diseases, for in that case they might obtain hints for treatment which would not otherwise be presented. In regard to the value of dental knowledge to the physician, Dr. J. Marion Sims holds the following language: "I wish that medical men generally could be better educated in reference to the effects of diseased teeth on the general health. We are all familiar with the fact that diseased teeth frequently cause neuralgia, and this is the extent of medical education on that point. They usually do not recognize the fact that, as a general thing, diseased teeth, with inflamed sockets, with matter exuding from them, are the cause of producing more numerous diseases, more *terrible consequences* to the general health, than almost any other cause that can occur. I could state cases in which some simple knowledge of this subject would enable the medical man to treat them more successfully than he does. It is a source of regret that medical men have so little knowledge of dental diseases."

A more general diffusion of dental knowledge in the minds of the masses is a want for their guidance in the care necessary for the preservation of their teeth. If there was more popular dental literature furnished the public than there has been, both the dentist and his patients would be benefitted. And it would not be amiss could the subject be made a part of school education. When the large amount of suffering that is endured from the lack of proper care on the part of individuals, and especially of the young, is considered, it would appear to be a prime necessity that they should be better instructed as to the requirements of those important organs, the teeth. To study the history of these pearly gems, from their earliest fœtal and highly vascular germ life, until they have assumed a permanent and indurated formation, is a subject that would be found of interest to many outside of the profession. To observe them assuming their infantile armor, one sufficient to contend with the pliant and delicate antagonisms to be met with in their brief existence, to the fully-panoplied adults, to war upon the stern realities of a long life, and nature, in her abundance, has no more interesting subject of study, for the lovers of her mysteries.

Camden, S. C. M. B.

